# Individual exploratory responses are not repeatable across time or context for four species of food-storing corvid

**DOI:** 10.1038/s41598-019-56138-y

**Published:** 2020-01-15

**Authors:** Alizée Vernouillet, Debbie M. Kelly

**Affiliations:** 1 Department of Biological Sciences, University of Manitoba, Winnipeg, Canada; 2 Department of Psychology, University of Manitoba, Winnipeg, Canada

**Keywords:** Psychology, Animal behaviour

## Abstract

Exploration is among one of the most studied of animal personality traits (i.e., individual-level behavioural responses repeatable across time and contexts). However, not all species show clear evidence of this personality trait, and this is particularly so for members of the Corvidae family. We assessed the exploratory behaviour of four food-caching corvid species: pinyon jays (*Gymnorhinus cyanocephalus*), Clark’s nutcrackers (*Nucifraga columbiana*), California scrub jays (*Aphelocoma californica*), and black-billed magpies (*Pica hudsonia*). Contextual repeatability was assessed through examining behavioural measures during the Novel Environment task and the Novel Object task, whereas temporal repeatability was assessed by examining changes in these measures over repeated trials. Our results suggest that, for corvids, an individual’s exploratory behaviour was not repeatable across contexts or over time. Hence, we found no evidence that exploration constitutes a personality trait for these species of corvid. We did find differences in exploratory behaviour, at a species level, that may be explained by relative reliance on cached food.

## Introduction

Animal personality has been defined as a suite of behaviours that differs among individuals but is repeatable across time and contexts within an individual^1–4^. For several decades, animal personality has been investigated using a wide range of species, including invertebrates, birds, mammals, reptiles, amphibians, and fish^2,5,6^. For non-human animals, five personality traits are often investigated: exploration, neophobia, aggressiveness, sociability, and activity^4,7^. These traits may have important implications at the individual level as well as at the species level, as they may influence the fitness of individuals^4,8^. For this reason, it is important to examine whether animals have repeatable behavioural responses to better understand how they cope with novel or changing environments.

Exploration of novel environments and novel objects allow individuals to learn about their social and physical surroundings, as well as encounter new resources^4,9^. However, exploration of novelty can also be associated with an increased risk of predation^4^. For this reason, an individual’s exposure to novelty may trigger two seemingly opposite, yet independent, responses within an individual: avoidance (i.e., neophobia) and approach or exploration^10,11^. Neophobia may interfere with exploration, because if an individual is avoiding novelty, the individual will have fewer opportunities to explore or interact with a novel stimulus^10^. To optimize the value gained from exploring novelty, individuals need to balance the potential benefit of exploring and the risks of increased predation. As animals are frequently exposed to novelty, and these experiences may be on the rise due to an increase in human-induced environmental changes^12^, it is becoming increasingly important to understand how individuals explore novelty to better evaluate whether these environmental changes affect species differently.

To assess an individual’s exploratory behaviour, two tasks have been commonly used for studies of non-human animals: the Novel Environment task and the Novel Object task (for a review see^13^). During the Novel Environment task, an individual is placed in an unfamiliar environment, and the number of movements made within the environment is viewed as a measure of spatial exploration^13,14^. During the Novel Object task, an individual is presented with a single novel object within a familiar environment (such as a home cage), and the latency to approach the object is viewed as a measure of object exploration^13,14^. Both tasks have been used to investigate whether an individual’s exploratory behaviour changes during its development^15^, or correlates with other personality traits^13^, fitness^8^ or cognitive abilities^16^.

To determine whether an individual’s exploratory behaviour is a personality trait, it has to be repeatable across contexts (i.e., contextual repeatability^17^) and over time (i.e., temporal repeatability). To investigate whether an individual’s exploratory behaviour is repeatable across different contexts, previous studies have administered the Novel Environment and the Novel Object tasks together to assess whether the main behaviours measured during these tasks correlate at an individual level^14,18–20^. Similarly, to investigate whether an individual’s exploratory behaviour is repeatable over time, previous studies have administered the tasks multiple times to assess the repeatability of the main behavioural measures at an individual level^21,22^. Using this approach, the presence of personality traits has been supported for multiple species (e.g., Eurasian blackbirds^23^, great tits^24–26^, mallards^27^, starlings^28^, zebra finches^29^). However, studies focusing on species within the Corvidae family have shown that exploratory responses may not be correlated across tasks^15,20^, nor repeatable over time^21^, suggesting instead that exploration may be context-dependent. Hence, investigating corvids’ exploratory behaviour, including whether it is context-dependent, provides us with an opportunity to understand the potential uniqueness of this bird family among other animal species.

Despite the growing number of studies focusing on animal personality, how and why different personalities evolved and persist remains unclear. Since an individual’s behavioural responses may be constrained across time and contexts, this apparent inflexibility can be maladaptive in some situations. However, researchers have suggested that this apparent inflexibility may be the result of a trade-off between current and future reproduction^30^ or an adaptation to a changing environment^31^, resulting in the presence of a great range of personalities within a population.

One avenue to gain insight into the evolutionary basis for the existence of animal personalities is to use a comparative approach^4,32^. Comparative studies allow researchers to isolate a factor that may explain why species differ in their behavioural responses. Yet, studies that have focused on the comparison of personality traits in related species remain limited (but see^32–35^). For this reason, we chose related species of North American corvid to assess the contextual and temporal repeatability of an individual’s exploratory behaviour. Pinyon jays (*Gymnorhinus cyanocephalus*) and Clark’s nutcrackers (*Nucifraga columbiana*) are two closely-related corvid species that rely heavily on thousands of cached pine nuts to survive through the winter^36^. Both species can be sympatric and display cache protection strategies when observed by conspecifics (Clark’s nutcrackers^37^, Clark’s nutcrackers and pinyon jays; Vernouillet *et al*., in prep). However, pinyon jays and nutcrackers differ in their relative sociality: pinyon jays are highly social, as they live in large flocks of hundreds of individuals^38^, whereas Clark’s nutcrackers are less social, as their social group usually comprises only the mating pair and their offspring of the year^39^. To further our comparative analyses, we also included results from a previously published study^20^ of two other related North American corvid species, black-billed magpies (*Pica hudsonia*) and California scrub jays (*Aphelocoma californica*), which participated in the same Novel Environment task^20^ as the pinyon jays and Clark’s nutcrackers in the current study. However, their relative sociality is intermediary in comparison to that of pinyon jays and Clark’s nutcrackers. Magpies’ social structure usually consists of non-territorial pairs that gather in small flocks^40,41^, and Western scrub jays form small flocks of semi-territorial mating pairs^40,42^. California scrub jays cache fewer food items, and rely on these caches for shorter periods of time^36,43^, compared to pinyon jays and Clark’s nutcrackers. Black-billed magpies, although a food-caching corvid, only cache items for a few hours to days, and are primarily considered to be an opportunistic omnivore^41,44,45^. All four species are year-round residents in North America^38–42^. Including data from these two species, in addition to those collected from Clark’s nutcrackers and pinyon jays in the current study, allowed us to more closely examine the contribution of relative sociality and feeding ecology to exploration in corvids.

The current study aimed to investigate the exploratory behaviour of corvids at the individual level and species level. At the individual level, we examined whether an individual’s exploratory behaviour constituted a personality trait. For this purpose, we assessed the temporal repeatability and the contextual repeatability of an individual’s exploratory behaviour. At the species level, we investigated whether corvids differed in their exploration by comparing the behavioural responses of four closely-related North American corvid species.

## Methods

### Subjects

Pinyon jays and Clark’s nutcrackers actively participated in this experiment (*n* = 12 for each species; seven female jays, six female nutcrackers). Additionally, we used previously published data^20^ from California scrub jays and black-billed magpies (*n* = 7 for each species; four female jays, three female magpies) housed in the same laboratory. Nutcrackers and both jay species were wild-caught as adults around Flagstaff (Arizona, USA), whereas magpies were wild-caught as pre-fledglings around Saskatoon (Saskatchewan, Canada) and hand-raised in captivity. All individuals were kept in captivity for approximately seven years, and had previous unrelated experiments (e.g., concept learning^46,47^; caching experiments^37,48^). All applicable international, national, and/or institutional guidelines for the care and use of animals were followed. Our research protocol was approved by University of Manitoba’s Animal Care Committee (#F2014-037) and complied with the guidelines set by the Canadian Council on Animal Care.

All individuals were housed in individual cages (jays: 51 × 51 × 72 cm, width x depth x height; nutcrackers and magpies: 82 × 54 × 76 cm), with multiple wood perches, at the University of Manitoba. Both jay species were held in the same colony room, whereas nutcrackers and magpies were held in a species-specific colony room. Colony rooms were maintained at 22 °C with a 12:12 day-night cycle, with light onset at 0700. Birds were given an ad libitum amount of water and grit during the study. Their regular diet consisted of parrot pellets, turkey starter, sunflower seeds, pine nuts, mealworms, peanuts, oyster shells, and a vitamin supplement Prime®.

The experimental procedures used during the Novel Environment task for the scrub jays and magpies were the same as reported here.

### Exploration tasks

To assess an individual’s exploratory behaviour, we used: the Novel Environment task and the Novel Object task (adapted from^14^). To measure the temporal repeatability of an individual’s exploratory behaviour, we conducted multiple trials of each task. During the first day, subjects received the first trial of the Novel Environment task. During days two through five, the subjects received four trials, one trial per day, of the Novel Object task. The second trial of the Novel Environment task was conducted six to eight months later. During the intermediary period, individuals participated in an unrelated caching experiment (i.e., pinyon jays and nutcrackers, Vernouillet *et al*., in prep) or in an inhibitory control experiment (i.e., scrub jays and magpies^20^), which was conducted in a room different from that used in the current study. Testing time of the day and order of individuals were randomized across species and individuals. All trials were video-recorded using an Everfocus® 1/3″ colour digital camera.

#### Novel environment task

Materials. Two different environments of the same size (221 × 312 × 200 cm, width × length × height) were used. All individuals experienced each environment in the same order. The size of the environments and the flooring material were similar to equate the amount of exploratory space and tactile experiences, respectively. To ensure the environments were perceived as novel, several visual properties differed between the two environments: one longer wall was covered by a cloth curtain in the first environment, whereas one shorter and two longer walls were covered by plastic curtains in the second environment. Five artificial trees made of laminate wood (61 × 86 cm, width × height), affixed to a wooden base^14,20^ were spaced evenly throughout the environment, and in the same locations for both environments. Each tree had four branches, comprised of perches used in the birds’ home cages. Birds were never exposed to the artificial trees nor to the environments prior to the current experiment.

Procedures. Individuals had ad libitum access to food prior to the start of each trial for at least 24 hours (maximum 30 hours), to control for food motivation. Within the experimental environment, a bird was placed in a wooden “start” box (31 × 38 × 32 cm, width × length × height) on the floor, along one of the shorter walls of the environment. To start a trial, the door of the start box was remotely opened. Birds were then permitted to move freely within the environment for a total of 20 minutes once the trial started. We did not force entry into the environment to better assess an individual’s self-motivated spatial exploration^13^.

Behavioural measures for the novel environment task (NE). For each trial four dependent measures were recorded: (1) NE Exit Latency – duration in seconds from when the start box door was opened to when the individual exited (i.e., when the bird’s entire body was out of the start box), (2) NE First Visit Latency – duration in seconds for an individual to visit the first tree (i.e., perching on at least one of a tree’s branches) from when it exited the start box, (3) NE Number of Trees – number of trees visited at least once, and (4) NE Number of Movements – number of “movements” made in the environment (i.e., individual flying or walking from one location to another, with a minimum of two seconds latency between movements). If an individual did not exit the start box within 20 minutes, it was given a NE Exit Latency of 1200 seconds; if an individual did not visit any of the trees within 20 minutes it was given a NE First Visit Latency of 1200 seconds and a NE Number of Trees of 0.

#### Novel object task

Materials. All trials were conducted in the same environment as used during the first trial of the Novel Environment task, and the start box remained in the same position. The birds experienced a total of four trials, each with a different object placed at the centre of the environment, and visible from the start box (Trial 1: red cup, Trial 2: green poker chip, Trial 3: black bottle, Trial 4: yellow plastic duck). The objects were selected to be distinctive, and small enough to allow individuals to interact with them. Only pinyon jays and nutcrackers performed this task.

Procedures. Birds were provided with ad libitum availability to food for at least 24 hours (maximum 30 hours) prior to the start of each trial to control for food motivation. Each trial started with the remote opening of the start box. Birds were permitted to move freely within the environment for 10 minutes. We did not force the entry into the environment nor place a food reward beside the novel object to better assess an individual’s interest to explore the object^13^.

Behavioural measures for the novel object task (NO). For each trial, three dependent measures were recorded: (1) NO Exit Latency – duration in seconds from the start of the trial to when the individual exited the start box (i.e., when the bird’s entire body was out of the start box), (2) NO Approach Latency – duration in seconds from when the individual exited the start box to when it approached within a radius of 50 cm of the novel object, and (3) NO Duration Close – relative duration spent less than 50 cm from the object (i.e., duration spent less than 50 cm from the object divided by duration spent outside of the start box [600 – NO Exit Latency]). If an individual did not exit the start box during a trial it was given a NO Exit Latency of 600 seconds; if a bird did not approach within 50 cm of an object, it was given a NO Approach Latency of 600 seconds and a NO Duration Close of 0.

### Analyses

All statistical analyses were conducted in R^49^ using *car*^50^, *dunn.test*^51^ and *rptR*^52^ packages. Alpha values of *p* < 0.05 were considered significant.

#### Individual responses to novelty

To assess whether dependent variables measured the same behaviour, we performed Spearman correlations between all measures collected within each task with a Bonferroni correction.

#### Temporal repeatability of an individual’s behavioural measures

To assess whether an individual’s behaviour during each task was repeatable, we ran one general linear mixed model for each behavioural measure collected to partition variance and calculated the adjusted repeatability (*R*) as the ratio of variance among individuals divided by total variance (individual plus residual variance) for each species using the *rpt* function of the *rptR* package^52^. We fit each model with a Poisson error structure since behavioural measures were count data and latencies and were not normally distributed. Models included trial number as a fixed effect and bird identity as a random effect^53^. We conducted parametric bootstrapping (*n* = 1,000 loops) to estimate 95% confidence intervals, and likelihood ratio tests to calculate *p* values for each repeatability estimate.

#### Contextual repeatability of an individual’s behavioural measures

To determine the contextual repeatability of an individual’s exploratory behaviour across tasks, we performed Spearman correlations between all repeatable measures using the data collected during the first trial of each task with a Bonferroni correction.

#### Behavioural variability at the species level

To compare the exploratory behaviours between corvid species, we performed Kruskal-Wallis tests on all variables for the Novel Environment task and Mann-Whitney tests on all variables for the Novel Object task. To compare the inter-individual variability between species, we performed Levene’s tests on all variables collected using the *car* package, and HSD Tukey’s tests for post-hoc comparisons using the *dunn.test* package.

## Results

### Overall species results

#### Novel Environment

During the first trial of the Novel Environment task, thirty-eight birds (n = 12 pinyon jays, n = 12 nutcrackers, n = 7 scrub jays, and n = 7 magpies) were tested. We found differences in the behavioural variables collected in the four corvid species (see Table 1 for all species comparisons using Kruskal-Wallis tests). Pinyon jays visited more trees than magpies (Dunn’s multiple comparison post-test: NE Number of Trees: *p* = 0.022) and nutcrackers made more movements than magpies (Dunn’s multiple comparison post-test: NE Number of Movements: *p* = 0.012).

**Table 1:** Behavioural measures collected during the Novel Environment task in four corvid species.

		Pinyon jays (M ± SE)	Clark’ nutcrackers (M ± SE)	Scrub jays (M ± SE)	Black-billed magpies (M ± SE)	*χ*	*p*
First trial	Exit Latency	24.2 ± 21.5	7.9 ± 2.1	146.4 ± 90.9	308.6 ± 171.6	7.33	0.062
	First Visit Latency	187.9 ± 120.1	328.6 ± 150.1	178.0 ± 170.2	731.6 ± 192.1	7.49	0.058
	Number of Trees	2.6 ± 0.6	2.3 ± 0.5	1.9 ± 0.7	0.4 ± 0.2	8.06	0.045*
	Number of Movements	24.2 ± 12.2	39.4 ± 11.2	17.7 ± 7.9	3.4 ± 1.3	8.38	0.039*
Second trial	Exit Latency	0.2 ± 0.1	343.0 ± 150.8	174.4 ± 170.9	423.8 ± 246.4	16.321	<0.001**
	First Visit Latency	244.4 ± 159.3	634.1 ± 176.4	343.9 ± 220.5	600.2 ± 248.8	6.627	0.085
	Number of Trees	1.7 ± 0.4	0.8 ± 0.5	1.3 ± 0.4	0.5 ± 0.2	5.573	0.134
	Number of Movements	5.3 ± 1.5	15.4 ± 4.9	3.0 ± 0.8	2.5 ± 1.2	5.499	0.139

Exit Latency, and First Visit were measured in seconds. Species comparisons were performed using Kruskal-Wallis tests. *p < 0.05, **p < 0.01.

Thirty-three birds (*n* = 10 pinyon jays, *n* = 10 nutcrackers, *n* = 7 scrub jays, and *n* = 6 magpies) participated in the second trial of the NE task. We found differences in the behavioural variables collected in the four corvid species (see Table 1 for all species comparisons). Pinyon jays had a lower latency to exit the start box than nutcrackers (Dunn’s multiple comparison post-test: NE Exit Latency: *p* = 0.001), scrub jays (Dunn’s multiple comparison post-test: NE Exit Latency: *p* = 0.013) and magpies (Dunn’s multiple comparison post-test: NE Exit Latency: *p* = 0.008).

#### Novel object

Twenty-three birds (*n* = 11 pinyon jays, and *n* = 12 nutcrackers) participated in all four trials of the NO task. We found some differences in the behavioural variables between the two corvid species (see Table 2 for all species comparisons using Mann-Whitney tests). Nutcrackers had a lower latency to exit the start box during the first trial, but pinyon jays had a lower latency to exit the start box during the second and third trials. Nutcrackers had a lower latency to approach the novel object during the third trial.

**Table 2:** Behavioural measures collected during the Novel Object task in two corvid species.

		Pinyon jays (M ± SE)	Clark’s nutcrackers (M ± SE)	*U*	*p*
First trial	Exit Latency	110.5 ± 73.0	45.8 ± 31.1	33.0	0.046*
	Approach Latency	280.3 ± 91.8	196.9 ± 76.9	76.0	0.557
	Duration Close	0.20 ± 0.03	0.11 ± 0.26	29.0	0.289
Second trial	Exit Latency	3.5 ± 1.7	107.2 ± 66.5	28.0	0.021*
	Approach Latency	257.9 ± 81.4	311.6 ± 76.0	70.5	0.805
	Duration Close	0.14 ± 0.29	0.02 ± 0.04	45.0	0.669
Third trial	Exit Latency	3.8 ± 0.8	160.5 ± 76.6	26.5	0.016*
	Approach Latency	546.8 ± 49.2	297.9 ± 85.5	113.5	0.004**
	Duration Close	0.00 ± 0.01	0.00 ± 0.01	33.0	0.384
Fourth trial	Exit Latency	56.9 ± 54.3	53.4 ± 49.7	45.0	0.208
	Approach Latency	278.6 ± 91.8	279.9 ± 84.9	67.5	0.951
	Duration Close	0.17 ± 0.31	0.07 ± 0.11	54.5	1.000

Exit Latency, and Approach Latency were measured in seconds. Duration Close was measured as a proportion. Species comparisons were performed using Mann-Whitney tests. *p < 0.05, **p < 0.01.

### Individual responses to novelty

#### Novel environment

For pinyon jays, NE Number of Trees and NE Number of Movements were positively correlated (*rho* = 0.84, *p* < 0.001; Table S1), indicating that individuals who made more movements in the environment tended to visit more trees. The NE Number of Trees and NE First Visit Latency tended to be negatively correlated (*rho* = −0.36, *p* = 0.096; Table S1), indicating that individuals who visited the first tree earlier in the trial tended to visit more trees. No other behavioural measures significantly correlated (Table S1).

For nutcrackers, NE Exit Latency positively correlated with NE First Visit Latency, indicating that individuals having a greater latency to exit the start box also had a greater latency to visit the first tree (*rho* = 0.45, *p* = 0.034; Table S2). Additionally, NE Number of Trees negatively correlated with NE First Visit Latency (*rho* = −0.63, *p* = 0.002) and positively with NE Number of Movements (*rho* = 0.55, *p* = 0.008; Table S2), indicating that individuals that visited more trees had a shorter latency to visit the first tree and made more movements. There was also a tendency for NE Number of Trees to be negatively correlated with NE Exit Latency (*rho* = −0.41, *p* = 0.056; Table S2), indicating that individuals who left the start box later in the trial tended to visit fewer trees. No other behavioural measures significantly correlated (Table S2).

For scrub jays, only NE First Visit Latency and NE Number of Trees tended to negatively correlate (*rho* = −0.52, *p* = 0.054; Table S3), indicating that individuals who visited the first tree earlier in the trial tended to visit more trees. No other behavioural measures correlated (Table S3).

For magpies, NE Exit Latency positively correlated with NE First Visit Latency (*rho* = 0.82, *p* < 0.001; Table S4) and negatively correlated with NE Number of Trees (*rho* = −0.65, *p* = 0.017; Table S4), indicating individuals that had a greater latency to exit the start box also had a greater latency to visit the first tree and visited fewer trees. NE First Visit Latency and NE Number of Trees negatively correlated (*rho* = 0.83, *p* < 0.001), indicating that individuals who visited the first tree earlier in the trial tended to visit more trees. No other behavioural measures correlated (Table S4).

#### Novel object

For pinyon jays, NO Duration Close was negatively correlated with NO Exit Latency (*rho* = −0.44, *p* = 0.005; Table S5) and with NO Approach Latency (*rho* = −0.82, *p* < 0.001; Table S5). Individuals that spent a proportionally longer duration close to the object had a shorter latency to exit the start box and a shorter latency to approach the object once out of the start box.

For nutcrackers, NO Approach Latency was negatively correlated with NO Exit Latency (*rho* = −0.36, *p* = 0.011; Table S6) and with NO Duration Close (*rho* = −0.76, *p* < 0.001; Table S6), indicating that individuals that had a shorter latency to approach the novel object had a longer latency to exit the start box and spent relatively more time close to the novel object.

### Temporal repeatability of an individual’s behavioural measures

#### Novel environment

**Figure 1:**
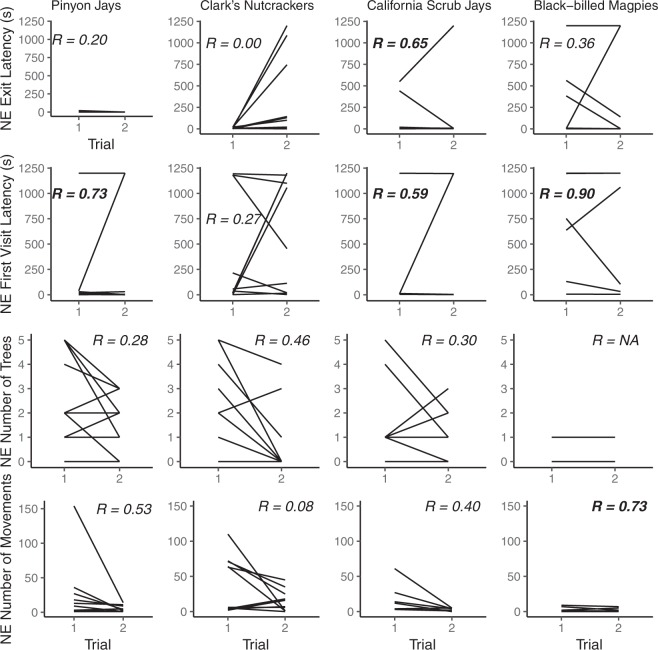
Individual behavioural differences for the four dependent variables collected during the Novel Environment task in the four corvid species. Each line represents one individual. Significant repeatability estimates are indicated in bold.

For individual pinyon jays, NE Exit Latency and NE Number of Trees were not repeatable across trials (NE Exit Latency: *n* = 10, *R* = 0.20 [95% CI: 0.00-0.67], *p* = 0.306; NE Number of Trees: *n* = 10, *R* = 0.28 [95% CI: 0.00–0.54, *p* = 0.149), but NE First Visit Latency was (NE First Visit Latency: *n* = 10, *R* = 0.73 [95% CI: 0.18–0.95], *p* = 0.011) and there was a tendency for NE Number of Movements to be repeatable over trials (NE Number of Movements: *n* = 10, *R* = 0.53 [95% CI: 0.00–0.92], *p* = 0.053; Fig. 1).

For individual nutcrackers, NE Exit Latency, NE First Visit Latency and NE Number of Movements were not repeatable across trials (NE Exit Latency: *n* = 10, *R* = 0.00 [95% CI: 0.00–0.69], *p* = 1.000; NE First Visit Latency: *n* = 10, *R* = 0.27 [95% CI: 0.00–0.78], *p* = 0.184; NE Number of Movements: *n* = 10, *R* = 0.08 [95% CI: 0.00–0.70], *p* = 0.407), but there was a tendency for NE Number of Trees to be repeatable across trials (NE Number of Trees: *n* = 10, *R* = 0.46 [95% CI: 0.00–0.73], *p* = 0.092; Fig. 1).

For individual scrub jays, NE Number of Trees and NE Number of Movements were not repeatable across trials (NE Number of Trees: *n* = 7, *R* = 0.30 [95% CI: 0.00–0.66], *p* = 0.249; Number of Movements: *n* = 7, *R* = 0.44 [95% CI: 0.00–0.92], *p* = 0.162), but NE Exit Latency and NE First Visit Latency were (NE Exit Latency: *n* = 7, *R* = 0.65 [95% CI: 0.00–0.97], *p* = 0.031; NE First Visit Latency: *n* = 7, *R* = 0.59 [95% CI: 0.00–0.96], *p* = 0.045; Fig. 1).

For individual magpies, NE Exit Latency was not repeatable across trials (NE Exit Latency: *n* = 6, *R* = 0.36 [95% CI: 0.00–0.92], *p* = 0.187), but NE First Visit Latency and NE Number of Movements were (NE First Visit Latency: *n* = 7, *R* = 0.90 [95% CI: 0.47–0.99], *p* < 0.001; NE Number of Movements: *n* = 6, *R* = 0.73 [95% CI: 0.00–0.91], *p* = 0.028; Fig. 1). Repeatability for NE Number of Trees was not calculated as individuals only visited zero or one tree.

#### Novel object

**Figure 2:**
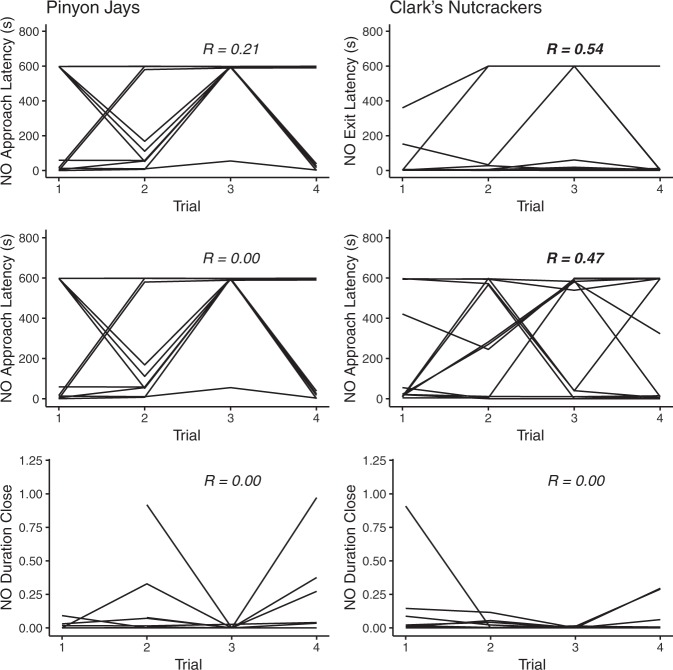
Individual behavioural differences for the three dependent variables collected during the Novel Object task in the two corvid species. Each line represents one individual. Significant repeatability estimates are indicated in bold.

For pinyon jays, NO Approach Latency and NO Duration Close were not repeatable across trials (NO Approach Latency: *n* = 11, *R* = 0.00 [95% CI: 0.00–0.28], *p* = 1.000; NO Duration Close: *n* = 11, *R* = 0.00 [95% CI: 0.00-1.00], *p* = 1.000), but there was a tendency for NO Exit Latency to be repeatable across trials (NO Exit Latency: *n* = 11, *R* = 0.21 [95% CI: 0.00–0.52], *p* = 0.082; Fig. 2).

For nutcrackers, NO Exit Latency and NO Approach Latency were repeatable over trials (NO Exit Latency: *n* = 12, *R* = 0.54 [95% CI: 0.16–0.78], *p* < 0.001; NO Approach Latency: *n* = 12, *R* = 0.47 [95% CI: 0.08–0.69], *p* < 0.001), but NO Duration Close was not (NO Duration Close: *n* = 12, *R* = 0.00 [95% CI: 0.00-1.00], *p* = 1.000; Fig. 2).

### Contextual repeatability of an individual’s behavioural measures

For pinyon jays, correlations between both tasks using repeatable variables were non-significant: 1) NE First Visit and NO Exit Latency (*rho* = 0.252, *p* = 0.454), and 2) NE Number of Movements and NO Exit Latency (*rho* = 0.030, *p* = 0.929).

For nutcrackers, correlations between repeatable variables between tasks were non-significant: 1) NE Number of Trees and NO Exit Latency (*rho* = 0.041, *p* = 0.899), and 2) NE Number of Trees and NO Approach Latency (*rho* = −0.266, *p* = 0.403).

### Behavioural variability at the species level

**Figure 3:**
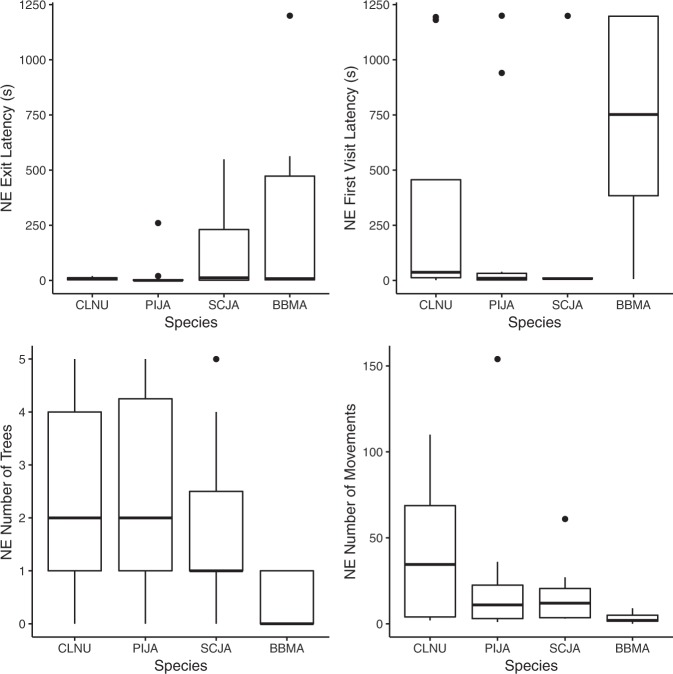
Inter-individual variability in four North American corvid species tested in the Novel Environment task: (**a**) NE Exit Latency, (**b**) NE Number of Movements, and (**c**) NE Number of Trees. BBMA: black-billed magpies, CLNU: Clark’s nutcrackers, PIJA: pinyon jays, and SCJA: California scrub jays.

During the first trial of the Novel Environment task, the four species also displayed different amount of variability within NE Exit Latency, NE Number of Movements, and NE Number of Trees, but not within NE First Visit Latency (NE Exit Latency: *F*_*3,34*_ = 14.67, *p* < 0.001; NE First Visit Latency: *F*_*3,34*_ = 0.66, *p* = 0.580; NE Number of Movements: *F*_*3,34*_ = 3.762, *p* = 0.020; NE Number of Trees: *F*_*3,34*_ = 4.118, *p* = 0.014; Fig. 3). The inter-individual variability in the latency to exit the start box was significantly smaller for pinyon jays and nutcrackers than for scrub jays and for magpies (Tukey’s HSD: nutcrackers-magpies: *p* < 0.001; pinyon jays-magpies: *p* < 0.001; nutcrackers-scrub jays: *p* = 0.010; pinyon jays-scrub jays: *p* = 0.041); whereas the inter-individual variability for the number of movements made in the novel environment was only significantly greater for nutcrackers compared to magpies (Tukey’s HSD: nutcrackers-magpies: *p* = 0.014). Finally, inter-individual variability in the number of number of trees visited was significantly greater for nutcrackers and pinyon jays than for magpies and tended to be greater for scrub jays than for magpies (Tukey’s HSD: nutcrackers-magpies: *p* = 0.028; pinyon jays-magpies: *p* = 0.012; scrub jays-magpies: *p* = 0.075).

During the first trial of the Novel Object task, we found no differences in the amount of variability for each behavioural variable between pinyon jays and nutcrackers (NO Exit Latency: *F*_*1,21*_ = 0.736, *p* = 0.401; NO Approach Latency: *F*_*1,21*_= 0.612, *p* = 0.443; NO Duration Close: *F*_*1,17*_ = 0.798, *p* = 0.384).

## Discussion

The goals of our study were: (1) to determine the temporal and contextual repeatability of exploratory behaviour in corvids at the individual level, using two commonly-used exploration tasks (Novel Environment and Novel Object), and (2) to compare exploration among corvid species. Generally, our overall results showed that an individual’s exploratory behaviour was not temporally repeatable. We also found that the behavioural measures of exploration at the individual level did not correlate between tasks, suggesting an individual’s exploratory behaviour was not contextually repeatable. Together, these results suggest that the behaviour assessed during the two tasks did not constitute a personality trait. However, we found some differences in the exploratory behaviour at the species level. During the Novel Environment task, magpies visited significantly fewer trees and made fewer movements than pinyon jays or nutcrackers, respectively. During the Novel Object task, pinyon jays and nutcrackers differed in their latency to exit the start box. Finally, inter-individual variability in exploratory behaviour significantly differed between magpies and pinyon jays, as well as between magpies and nutcrackers.

Across four corvid species, we investigated whether an individual’s exploratory behaviour might be considered as a personality trait. Specifically, we investigated the temporal and contextual repeatability of an individual’s exploratory behaviour, by examining behaviour across repeated trials for two different tasks. Our results showed that only some behavioural variables were repeatable over time for each task and for each species. Particularly, the latency to visit the first tree was repeatable for three of the four corvid species during the Novel Environment task, and may be considered partial evidence supporting a personality trait. However, overall, the lack of repeatability may also be partially explained by reduced novelty, as with repeated exposure the environment, novelty is necessarily reduced. As individuals became more familiar with the environment, the number of movements they made and the number of trees they visited decreased^54,55^. The species studied all are food-storing corvids with strong spatial memory capabilities, therefore it may be expected that these species would reduce exploration due to environmental familiarity. Thus, behavioural variables examined during subsequent trials may not accurately represent an individual’s exploratory behaviour in more novel envrionments^13,56^.

We found no contextual repeatability for the behavioural responses made by individual pinyon jays or nutcrackers. We also did not find any significant correlations among the behavioural measures between the two tasks. This result indicated the lack of stability of an individual’s rank order relative to others. Overall, we found no evidence that individuals’ exploratory behaviours were repeatable across contexts for pinyon jays and Clark’s nutcrackers. These results support the idea that, instead, an individual’s exploratory behaviour may be context-specific in corvids^20,21,57^ (as has been shown for other species^58–60^).

Another possible explanation for our failure to find individual level temporal and contextual repeatability for exploratory behaviour may be that behavioural measures examined during these tasks were assessing different aspects of personality or that a combination of personality traits may be contributing to the measures; the measurements are not selective for only exploratory behaviour^60,61^. During our study, this explanation was supported by the fact that most of the behavioural measures examined did not correlate within a task. The Novel Environment task has been previously used to assess not only exploration, but also activity and neophobia, whereas the Novel Object task has been used to assess neophobia in addition to exploration^13,62,63^. By controlling for food motivation and not forcing the birds to enter into the novel environment, we aimed to limit the influence of neophobia on the behavioural measures we assessed^13^. Instead, the behavioural measures used to assess the approach and interaction an individual has with a novel object (i.e., exploration), was not confounded by the hunger state of the individual^13^. However, these manipulations may not have completely removed the influence of neophobia and other personality traits such as activity on the individuals’ behaviour. For instance, it is possible that measures, such as the number of movements an individual made, or the number of trees visited in a novel environment, may measure a combination of an individual’s exploratory and activity behaviour^13^. An individual’s latency to exit the start box and latency to approach the novel object, or visit the first tree, may be the combination of both neophobia and exploration^10^. Hence, the dependent variables collected during both tasks might assess a behaviour that depends on different personality traits rather than an intrinsic exploratory behaviour. For this reason, and as suggested by previous studies^59,61^, we stress that future research should attempt to validate the tasks used or the study of animal personality.

Species comparisons provide opportunities to examine the variability of behavioural responses at the level of a species^4^. Comparative studies of variability in individual’s behavioural responses across species are limited^33,34,64–69^, even though this approach is necessary to identify potential factors contributing to individual-level variability of behavioural responses among closely-related species. Previous comparative studies that focused on identifying factors influencing exploratory behaviour at a species level have identified three main factors: the relative sociality of the species (e.g., Social Niche Hypothesis^70,71^), the foraging ecology and habitat complexity of the species^10,33,34,72^, and the migratory patterns of the species^67,73^. During our study, we compared the exploratory behaviour among four corvid species, which differed in relative sociality and foraging ecology. Pinyon jays are highly social, magpies and scrub jays are intermediary, and Clark’s nutcrackers are less social^44^. Pinyon jays and Clark’s nutcrackers are both specialist foragers and rely on long-term caching of pine nuts to endure periods lacking resources^38–40^, scrub jays are generalists that rely relatively less on food caches, and finally magpies are opportunist omnivores that only cache food for hours to days^36,41–45^. Although we did not directly examine the influence of sociality and foraging ecology on the exploratory behaviour of our four corvid species, this study provides a first insight into the factors underlying potential behavioural differences among these corvids.

The Social Niche Hypothesis stipulates that group-living individuals may maintain stable social roles (i.e., social niches) to reduce conflicts with other group members over resources^70,71^. Consequently, more social species should have a greater number of social niches and greater inter-individual variability in personalities than less social species so that individuals do not overlap in their social roles. We found no significant differences in the inter-individual variability of the highly-social pinyon jays and less social nutcrackers. Additionally, black-billed magpies and scrub jays had greater individual-level variability in their latency to enter the novel environment than pinyon jays and nutcrackers. On the contrary, nutcrackers, pinyon jays and scrub jays had a greater inter-individual variability in the exploratory measures compared to magpies. Thus, although we found significant species differences in the inter-individual variability of exploratory responses, these differences do not align with the relative level of sociality of each species. However, this pattern may better align with the species’ relative reliance on food-storing – their foraging ecology. The two species that rely most on cached food (i.e., pinyon jays and Clark’s nutcrackers) did not differ in their inter-individual variability in the exploratory measures, but both significantly differed from black-billed magpies, which rely less on cached food. Furthermore, California scrub jays, showed intermediate exploratory behaviour, as would be predicted by their foraging ecology. Additionally, our results showed that pinyon jays were faster to exit the start box and visited more trees than magpies, and nutcrackers made more movements than magpies. This pattern of species-level exploratory behaviour by food-caching corvids contradicts previous studies showing that generalist species are more exploratory than specialist species^34,68,74^. Instead, our results lend support to the idea that species relying on seasonal food resources are more exploratory^11,33^. Future research is necessary to determine whether corvids generally show different patterns of exploratory behaviour, compared to other birds, or if cache-reliance is driving these differences.

Other potential explanations exist for the differences in the exploratory behaviour between magpies and the other corvid species we examined. Perhaps the most interesting may be related to the rearing environment of the birds. The magpies were hand-raised, whereas the three other species were wild-caught as adults. To our knowledge, the influence of developmental experiences on exploratory behaviour has not yet been examined in related corvid species. Thus, whether the pattern of results we found are affected by these early experiences will require future investigation.

In summary, the current study assessed the repeatability of an individual’s exploratory behaviour during two commonly-used tasks for four species of corvid. Our results support recent findings that exploration is context-dependent. Additionally, we showed that an individual’s exploratory behaviour as measured in these tasks does not seem to constitute a personality trait, as the temporal and contextual repeatability of the behavioural variables collected during the two tasks was low. We recommend that future research focus on validating tasks used to assess an individual’s exploratory behaviour. We also suggest that future studies focus on the context-dependency of exploratory behaviour by corvids. At a species level, we found no supporting evidence that relative sociality, across closely-related species, explains inter-individual variability in exploration. Instead, at least for corvids, our results tend to support foraging ecology as an explanatory factor driving exploration.

## Supplementary information

Supplementary Information

Supplementary information

## Data Availability

Data has been made available as Supplementary Material.
