# Supplementary material for: Individual exploratory responses are not repeatable across time or context for four species of food-storing corvid
Source: Sci Rep. 2020 Jan 15;10:394. doi: 10.1038/s41598-019-56138-y (PMC6962162; doi:10.1038/s41598-019-56138-y)
Supplement: Supplementary file 2 — Supplementary information [file 41598_2019_56138_MOESM2_ESM.docx]

Individual exploratory responses are not repeatable across time or context for

four species of food-storing corvid

Alizée Vernouillet^1^ & Debbie M. Kelly^1,2*^

1. Department of Biological Sciences, University of Manitoba, Winnipeg, Canada
2. Department of Psychology, University of Manitoba, Winnipeg, Canada

Supplementary Material

Table S1: Spearman’s correlation matrix between the behavioural measures collected during the Novel Environment task in pinyon jays (*n* = 12). P-values are indicated between brackets: * *p* < 0.05, ** *p* < 0.01.

| Pinyon jays | NE First Visit Latency | NE Movements | NE Trees |
| --- | --- | --- | --- |
| NE Exit Latency | -0.10 (0.664) | -0.31 (0.158) | -0.33 (0.140) |
| NE First Visit Latency |  | -0.08 (0.708) | -0.36 (0.096) |
| NE Movements |  |  | 0.84 (<0.001**) |

Table S2: Spearman’s correlation matrix between the behavioural measures collected during the Novel Environment task in nutcrackers (*n* = 12). P-values are indicated between brackets: * *p* < 0.05, ** *p* < 0.01.

| Nutcrackers | NE First Visit Latency | NE Movements | NE Trees |
| --- | --- | --- | --- |
| NE Exit Latency | 0.45 (0.034*) | -0.09 (0.681) | -0.41 (0.056) |
| NE First Visit Latency |  | 0.09 (0.706) | -0.63 (0.002**) |
| NE Movements |  |  | 0.55 (0.008**) |

Table S3: Spearman’s correlation matrix between the behavioural measures collected during the Novel Environment task in California scrub jays (*n* = 7). P-values are indicated between brackets: * *p* < 0.05, ** *p* < 0.01.

| Pinyon jays | NE First Visit Latency | NE Movements | NE Trees |
| --- | --- | --- | --- |
| NE Exit Latency | 0.20 (0.485) | -0.42 (0.138) | -0.18 (0.548) |
| NE First Visit Latency |  | 0.24 (0.412) | -0.52 (0.054) |
| NE Movements |  |  | 0.24 (0.402) |

Table S4: Spearman’s correlation matrix between the behavioural measures collected during the Novel Environment task in black-billed magpies (*n* = 7). P-values are indicated between brackets: * *p* < 0.05, ** *p* < 0.01.

| Nutcrackers | NE First Visit Latency | NE Movements | NE Trees |
| --- | --- | --- | --- |
| NE Exit Latency | 0.82 (<0.001**) | -0.37 (0.214) | -0.65 (0.017*) |
| NE First Visit Latency |  | -0.38 (0.196) | 0.83 (<0.001**) |
| NE Movements |  |  | 0.42 (0.155) |

Table S5: Spearman’s correlation matrix between the behavioural measures collected during the Novel Object task in pinyon jays (*n* = 11). P-values are indicated between brackets: * *p* < 0.05, ** *p* < 0.01.

| Pinyon jays | NO Approach Latency | NO Duration Close |
| --- | --- | --- |
| NO Exit Latency | -0.17 (0.282) | -0.44 (0.005**) |
| NO Approach Latency |  | -0.82 (<0.001**) |

Table S6: Spearman’s correlation matrix between the behavioural measures collected during the Novel Object task in Clark’s nutcrackers (*n* = 12). P-values are indicated between brackets: * *p* < 0.05, ** *p* < 0.01.

| Pinyon jays | NO Approach Latency | NO Duration Close |
| --- | --- | --- |
| NO Exit Latency | -0.36 (0.011*) | -0.10 (0.534) |
| NO Approach Latency |  | -0.76 (<0.001**) |
